# Germline and somatic variants in ovarian carcinoma: A next-generation sequencing (NGS) analysis

**DOI:** 10.3389/fonc.2022.1030786

**Published:** 2022-12-01

**Authors:** Angeliki Andrikopoulou, Eleni Zografos, Kleoniki Apostolidou, Anastasios Kyriazoglou, Alksistis-Maria Papatheodoridi, Maria Kaparelou, Konstantinos Koutsoukos, Michalis Liontos, Meletios-Athanasios Dimopoulos, Flora Zagouri

**Affiliations:** Department of Clinical Therapeutics, Alexandra Hospital, School of Medicine, National and Kapodistrian University of Athens, Athens, Greece

**Keywords:** ovarian cancer, next-generation sequencing, NGS, germline mutations, BRCA, HRD

## Abstract

**Background:**

Germline BRCA1/2 mutations are identified in 13-15% of ovarian cancers, while an additional 5-7% of ovarian cancers harbor somatic BRCA1/2 mutations. Beyond these mutations, germline or somatic aberrations in genes of the homologous recombination (HR) pathway such as *RAD51B/C/D, PALB2, ATM, BRIP1* may confer an HR deficiency in up to 50% of ovarian tumors. Next-generation sequencing (NGS) is a high-throughput massive parallel sequencing method that enables the simultaneous detection of several mutations in entire genomes.

**Methods:**

We performed NGS analysis in 86 patients with ovarian cancer treated in the Oncology Department of Alexandra University Hospital in order to identify the molecular landscape of germline and somatic mutations in ovarian cancer.

**Results:**

The genes with the highest number of pathogenic somatic mutations in high grade serous carcinoma (HGSC) patients were TP53 [68%; 34/50] and BRCA1 [22%; 11/50] followed by somatic mutations in RB1 [2%; 1/50], NF1 [2%; 1/50], BRCA2 [2%; 1/50], AKT1 [2%; 1/50], RAD50 [2%; 1/50], PIK3CA [2%; 1/50] genes. Of note, the most common TP53 genetic polymorphism was c.524G>A p.Arg175His in exon 5. Variants of unknown significance (VUS) detected in HGSC included ROS1 [26%; 13/50], RAD50 [6%; 3/50], BRCA2 [6%; 3/50], NOTCH1 [6%; 3/50], TP53 [6%; 3/50], AR [6%; 3/50]. As for germline mutations, BRCA1 [8/30; 27%] and BRCA2 [4/30; 13%] were the most common genes bearing pathogenic alterations in HGSC, while VUS germline mutations commonly affected HRR-related genes, including ATM (c.7816A>G), BRIP (c.2327 C>A), CHEK2 (c.320-5T>A).

**Conclusion:**

Overall, genetic testing should be offered in most patients with ovarian cancer to identify mutations in HRR genes and determine the population that would be susceptible to poly ADP ribose polymerase (PARP) inhibitors.

## Introduction

Ovarian cancer is the eighth leading cause of cancer-related mortality in women accounting for over 200 000 deaths in 2020 (1). Although advances have been made during the last years in ovarian cancer diagnosis and treatment, survival rates have changed modestly for decades. Five-year survival is still as low as 39% and 17% for patients with IIIC/IV disease respectively (2). Ovarian cancer remains a significant burden of morbidity and mortality globally with rising incidence in developing countries and increasing rates in high income countries due to population aging. Histologically, ovarian cancer is classified into two main subtypes: epithelial ovarian cancer (EOC) accounting for more than 95% of the cases and other nonepithelial subtypes, mostly germ cell and sex-cord stromal cancers (3). EOC is further subdivided in high-grade serous ovarian carcinoma (HGSC) which is the most common subtype [75%] and low-grade serous carcinoma (LGSC) [10%], endometrioid (EC) [10%], clear cell carcinoma (OCCC) [5%] and mucinous [2.4%] (3). Debulking surgery either upfront or interval after receiving three cycles of platinum-based chemotherapy remains the cornerstone of ovarian cancer treatment. Platinum-based first line chemotherapy consists the standard treatment for ovarian cancer patients following debulking surgery. After 2018 that GOG-0218 (NCT00262847) Phase III trial published its first results, antivascular agent Bevacizumab has been incorporated in the first-line treatment of patients with stage III/IV EOC (4). Currently, PARP inhibitors have been established as maintenance treatment after primary treatment with platinum-based chemotherapy initially in BRCA1/2 – mutated high-grade serous carcinoma stage III/IV and recently in combination with bevacizumab in homologous-recombination deficient (HRD) HGSC tumors (5, 6).

Molecular testing has been established for the treatment optimization of ovarian cancer but also for primary prevention and genetic counseling. As known, 40% - 60% of BRCA1 and 11% - 27% of BRCA2 germline carriers develop ovarian cancer. PARP inhibitors have been approved for the maintenance treatment of patients with BRCA1/2 somatic mutations. Apart from the well-known BRCA1/2 mutations, other mutations in homologous recombination repair (HRR) genes such as RAD51, ATM, ATR, BRIP1, PALB2, RB1, NF1, CDKN2A confer homologous recombination deficiency and increased susceptibility to PARP inhibitors. HGSC is associated with lower prevalence but recurrent somatic mutations in NF1, BRCA1, BRCA2, RB1, and CDK12 in around 5–8% of tumours (3). Myriad HRD test is a next generation sequencing-based (NGS) test that assesses the qualitative detection and classification of single nucleotide variants, insertions and deletions, and large rearrangement variants in protein coding regions and intron/exon boundaries of the BRCA1 and BRCA2 genes and the determination of Genomic Instability Score (GIS) which is an algorithmic measurement of Loss of Heterozygosity (LOH), Telomeric Allelic Imbalance (TAI), and Large-scale State Transitions (LST) on formalin-fixed paraffin embedded (FFPE) tumor tissue specimens. HR deficiency is estimated to affect nearly 50% of HGSC and confer benefit from the treatment with PARP inhibitors Olaparib and Niraparib that are approved for this population (7). Other frequently identified mutations include mutations in the PI3K/Akt signaling pathway in OCCC [40%] and EC [40%], in AT-rich interaction domain 1A (ARID1A) gene in OCCC [40-67%] and EC [30%] while mutations in TP53 are a nearly universal characteristic of HGSC [97%] (3). It becomes apparent that molecular testing is currently necessary to guide treatment options and personalize treatment.

Next-generation sequencing (NGS) is a massively parallel sequencing technology that captures a broad spectrum of mutations in entire genomes or targeted regions of DNA or RNA. This method enables the simultaneous analysis of several genes or gene regions with a single test compared to traditional methods. Next-generation sequencing could detect quickly and accurately mutations in ovarian cancer patients that could serve as druggable targets. We here performed NGS analysis in patients with ovarian cancer treated in the Oncology Department of Alexandra University Hospital. We aim to identify the molecular landscape of pathogenic germline and somatic mutations as well as mutations of uncertain significance (VUS) in ovarian cancer patients as determined by powerful NGS analysis.

## Materials and methods

86 women with histologically confirmed ovarian carcinoma that underwent NGS in paraffin blocks and blood samples during the period 10/2019 through 12/2021 were considered eligible for our study. All women were treated at the Oncology Department of “Alexandra” Hospital, Medical School, University of Athens, Greece. Our institution has been certified by the European Society of Gynecologic Oncology (ESGO) as a center of excellence for the treatment of ovarian cancer. Clinicopathological characteristics including age at diagnosis, stage of the disease, histological subtype, grade, performance of PDS or IDS, debulking status, performance status (ECOG), maintenance treatment, progression-free survival and overall survival were collected from the medical records of the patients. This study was approved by the Institutional Review Board of the Alexandra General Hospital of Athens and performed in accordance with the ethical standards described in the Declaration of Helsinki. An informed consent form was obtained from each of the eligible patients.

### FFPE DNA sequencing

Formalin-fixed paraffin-embedded (FFPE) ovarian cancer tissues derived from hysterectomy/bilateral salpingo - oophorectomy were analyzed. Paraffin-embedded ovarian specimens were cut at slices of 10μm diameter. DNA was extracted from paraffinized tissue using the QIAamp Tissue kit (Qiagen, Germantown, MD, USA)and libraries were constructed using an Ion AmpliSeq Custom Next Generation Sequencing (NGS) DNA panel (Thermo Fisher Scientific, Waltham, MA, USA)., for the 58 targeted genes that are presented in **Supplementary Table 1**. The genotyping was performed using the Ion Torrent platform (Ion S5) since the automated amplicon-based library is prepared rapidly, enabling a fast turnaround time and a high degree of sample multiplexing, obtaining an average amplicon coverage of 500x for 91.39% of the targeted regions. We evaluated predicted pathogenic mutations, based on combined variant characterization by IonReporter (v5.12) (Thermo Fisher Scientific, Waltham, MA, USA).An additional manual inspection was performed using data from OncomineReporter (v4.4) and relevant databases (ClinVar, dbSNP, Ensemble, COSMIC, CIVIC, PharmGKB, OMIM, My Cancer Genome, VarSome etc).

### Germline DNA sequencing

Plasma blood samples were collected in Vacutainer tubes. Subsequently, genomic DNA was isolated from whole blood using the QIAsymphony DSP DNA Mini Kit (Qiagen, Germantown, MD, USA) and used to prepare indexed libraries using the Trusight Cancer Panel – Nextera DNA Flex Pre-Enrichment Library Prep (Illumina, San Diego, USA). Libraries were sequenced on a MiSeq genetic analyzer (Illumina, Inc., San Diego, CA), according to the manufacturer’s protocols. Annotation was performed against the human reference genome GRCh38 using VariantStudio V.3 (Illumina, Inc., San Diego, CA). Based on the data generated from this software, alterations were identified as pathogenic when classified as disease causing or as variants of unknown significance (VUS) when evidence regarding their pathogenicity was either conflicting or limited. The validation of results was performed according to criteria of American College of Medical Genetics – ACMG (8) and National Comprehensive Cancer Network (NCCN) guidelines.

### Statistical analysis

Continuous variables were summarized with the use of descriptive statistical measures [median and percentiles (25^th^,75^th^)] and categorical variables were displayed as frequency tables (N, %). The outcome of the debulking surgery was classified as optimal (residual disease below 1cm) or suboptimal. Overall Survival (OS) was defined as the time between the start of chemotherapy and the date of death from any cause. Progression-free Survival (PFS) was defined as the time between the start of chemotherapy and the date of disease progression. Alive patients were censored at the date of last contact. Kaplan-Meier estimates were used to describe and visualize the effect of categorical variables on OS and PFS. All statistical analyses were performed using the STATA/SE 16.0 software (Copyright 1985-2019 StataCorp LP).

## Results

### Patients baseline characteristics

Overall, 86 ovarian cancer patients underwent somatic and/or germline NGS analysis. Among them, there were 66 cases [76.7%] of high-grade serous carcinoma [HGSC], 7 cases [8.1%] of low grade serous carcinoma [LGSC], 7 cases [8.1%] of endometrioid carcinoma [EC], 4 cases [4.7%] of clear cell carcinoma [OCCC] and two cases [2.3%] of mucinous carcinoma.

Mean age at diagnosis was 58.67 (SD; 11) years for HGSC, 54.43 (SD; 11.73) years for LGSC and 51.29 (SD; 8.46) years for EC. Most patients were diagnosed at an advanced stage: IIIB [5; 5.8%], IIIC [36; 41.9%] and IV [20; 23.3%]. Primary debulking surgery was performed in 67 [77.9%] of cases, while 19 [22.1%] could not be upfront debulked and performed an interval debulking surgery. Debulking was optimal in the majority of cases [66; 76.7%] while it was suboptimal in [18; 20.9%]. 76 [88.4%] patients had performance status ECOG 0. Most of the patients received first line maintenance treatment with bevacizumab either alone [27; 31.4%] or in combination with PARP inhibitors [5; 5.8%], immune checkpoint inhibitors [4; 4.7%] or both of them [4; 4.7%].

### Survival analysis

**Figure 1:**
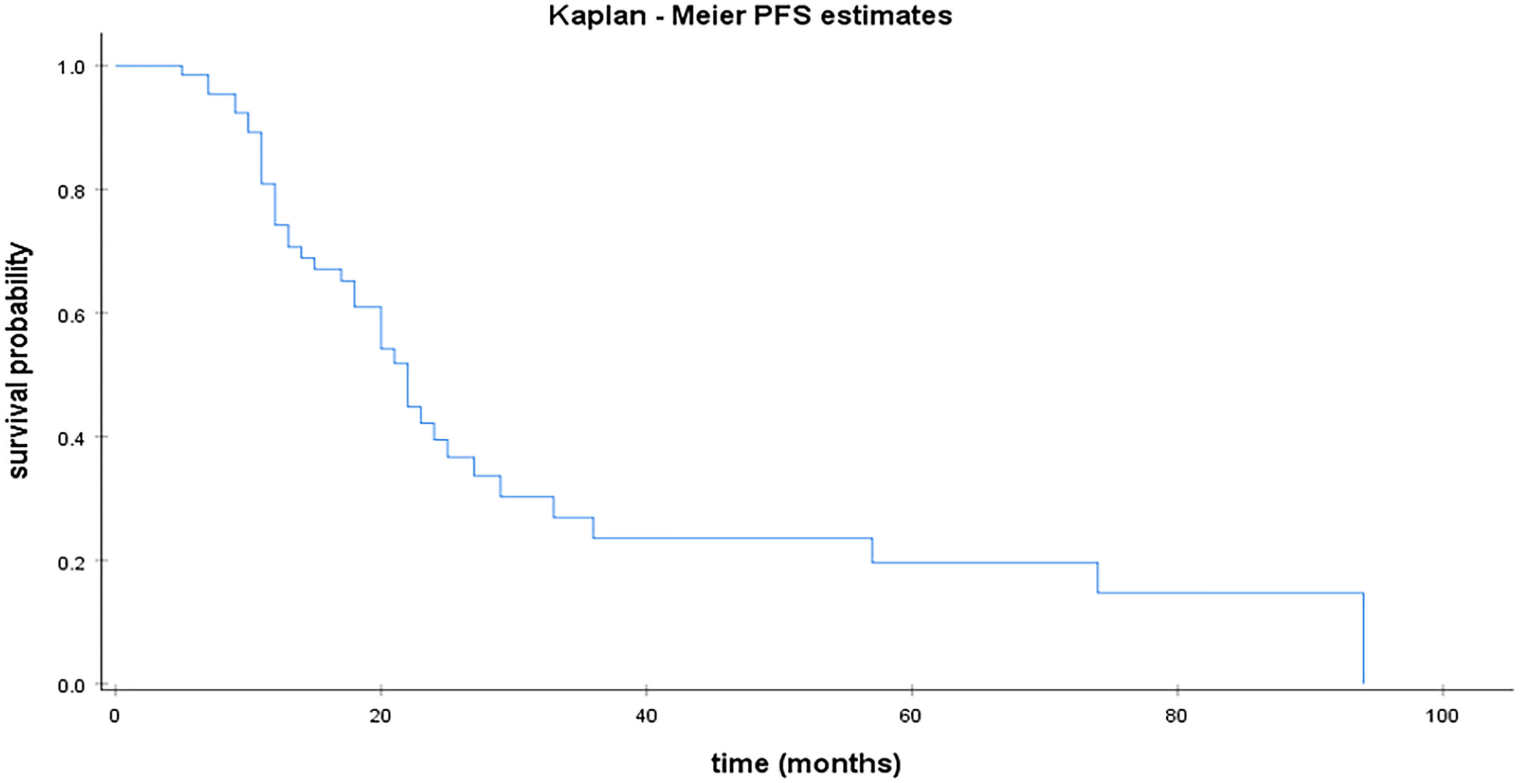
Kaplan Meier curve for PFS in HGSC.

**Figure 2:**
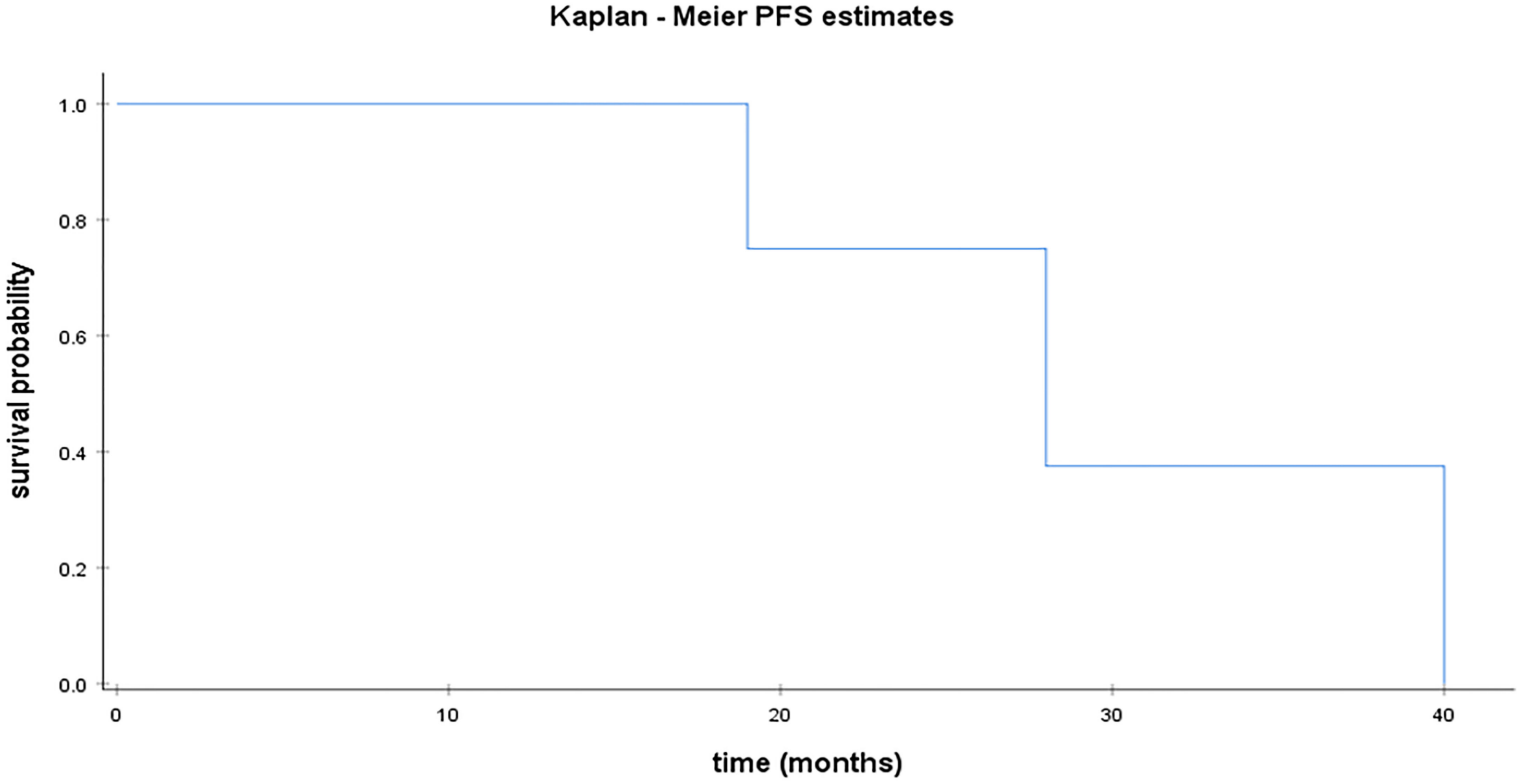
Kaplan Meier curve for OS in HGSC.

**Figure 3:**
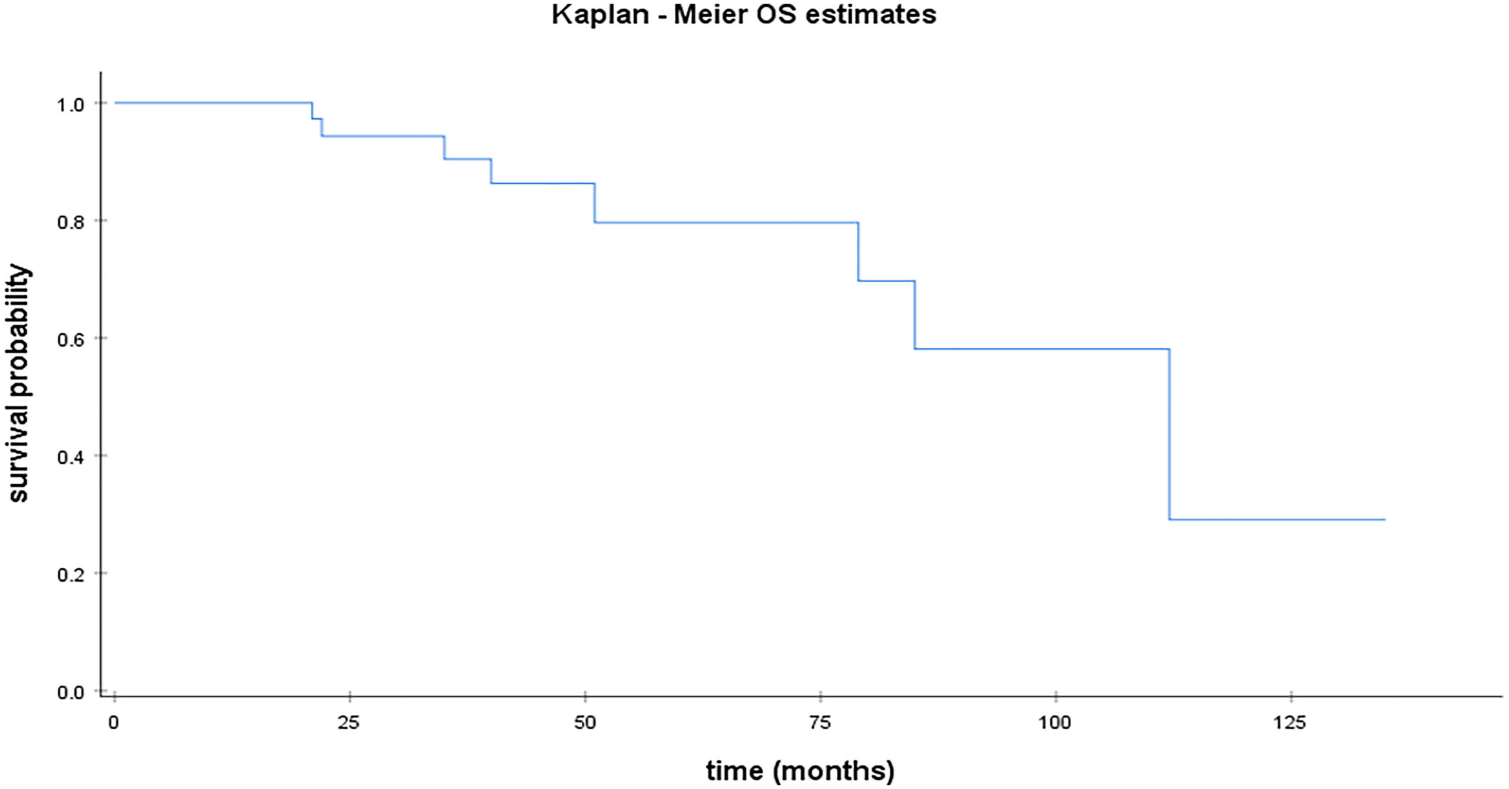
Kaplan Meier curve for PFS in LGSC.

Median PFS was 22 months for HGSC [95% CI; 18.57 – 25.42]. **Figure 1** illustrates median PFS in HGSC patients. For the remaining population, median PFS was 28 months [95% CI;14.52 – 41.47] for LGSC (**Figure 3**) and 44 months for EC. Median OS was 112 months [95% CI; 72.28 – 151.71] for patients with HGSC (**Figure 2**).

### HGSC

**Figure 4:**
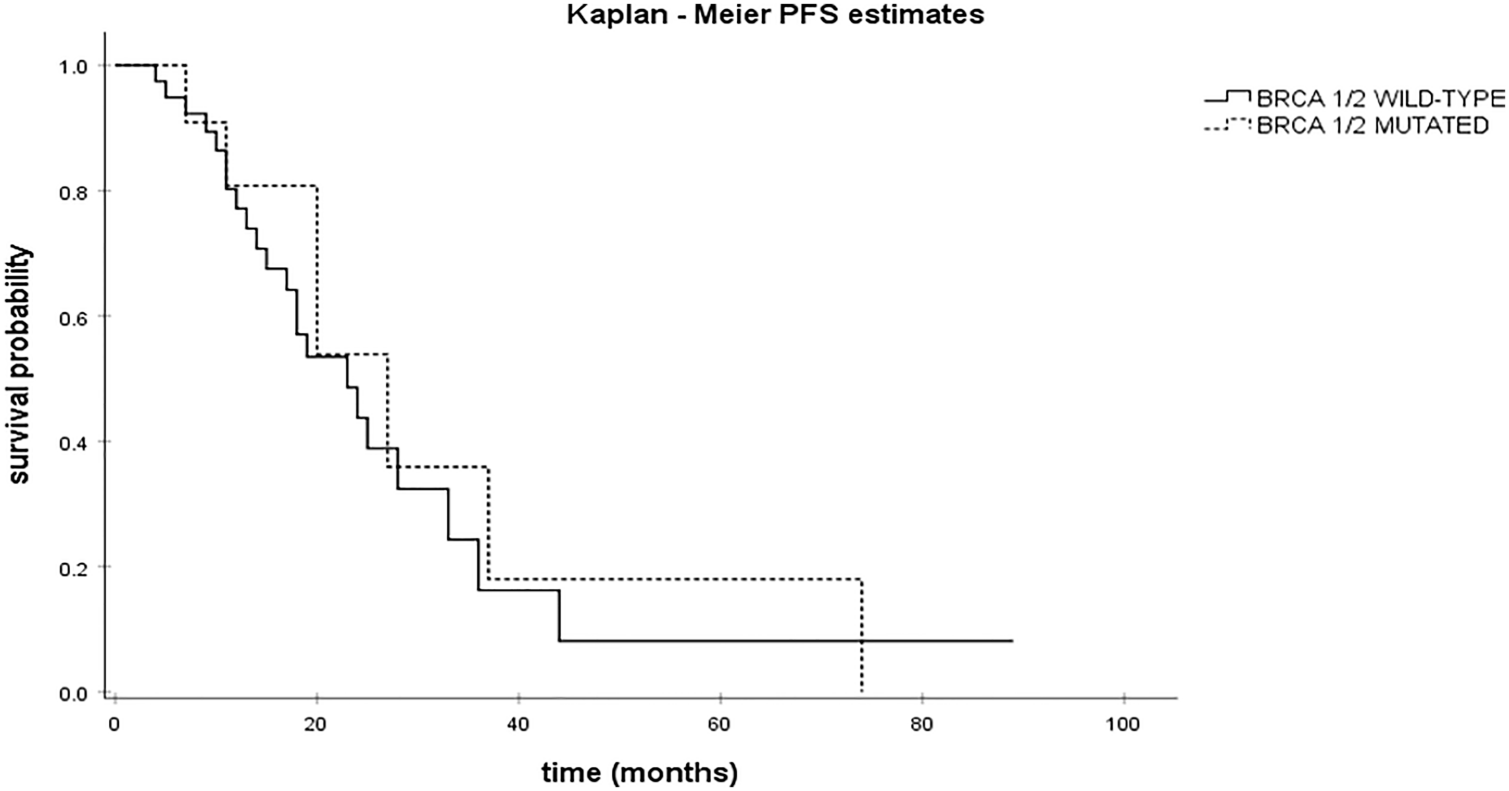
Kaplan Meier curves for PFS in BRCA1/2-mutated vs BRCA1/2 wild-type HGSC.

From 66 patients with HGSC, NGS analysis for somatic mutations was performed in 50 patients. All somatic mutations identified are presented in **Supplemental Table 3**. Overall, TP53 [68%; 34/50] and BRCA1 [22%; 11/50] were the genes with the highest number of pathogenic somatic mutations (**Table 1**) as expected. Of note, the most common TP53 genetic polymorphism was c.524G>A p.Arg175His in exon 5. Other genes bearing pathogenic somatic mutations were RB1 [2%; 1/50], NF1 [2%; 1/50], BRCA2 [2%; 1/50], AKT1 [2%; 1/50], RAD50 [2%; 1/50], PIK3CA [2%; 1/50]. Additional analysis was performed to explore the prognostic value of BRCA1/2 mutations (**Figure 4**). There was a trend for longer PFS in patients with BRCA1/2 mutations (27 months [95% CI; 13.89 – 40.10] vs 23 months [95% CI; 14.68 – 31.31]) although this association was not statistically significant (*p=0.65)*.

**Table 1:** Pathogenic somatic mutations identified in patients with HGSC.

Gene mutation	Genetic polymorphism	NCBI Genomes Browser	Frequency
TP53	exon8: c.818G>A p.Arg273His		1
	exon7: c.764_766del: p.Ile255del		1
	exon5: c.517G>A: p.Val173Met		1
	c.586C>T: p.Arg196*) chr17: 7578263G>A (hg19)	rs397516435	1
	c.614A>G p.Tyr205Cys chr17: 7578235 T>C	rs1057520007	1
	exon7: c.734G>T: p.Gly245Val		1
	exon5: c.524G>A p.Arg175His chr17: 7578406C>T	rs28934578	3
	exon8: c.840A>C: p.Arg280Ser		1
	c.796G>C: p.Gly266Arg chr17: 7577142C>G	rs1057519990	1
	c.1024del: p.Arg342Glufs*3 chr17: 7574003del		1
	exon6: c.659 A>G: p.Tyr220Cys		1
	c.673-1G>A chr17:7577609 C>T	rs878854073	1
	exon7: c.742 C>T: p.Arg248Trp		1
	exon8: c.833 C>T: p.Pro278Leu		1
	c.637 C>T: p.Arg213* chr17: 7578212 G>A	rs397516436	1
	exon4: c.245del: p.Pro82ArgfsTer41		1
	exon6: c.658T>A: p.Tyr220Asn		1
	exon5: c.438G>A: p.Trp146Ter		1
	exon7: c.742del: p.Arg248GlyfsTer97		1
	exon8: c.818G>A p.Arg273His		1
	exon10: c.997delC p.Arg333ValfsTer12		1
	c.541C>T p.Arg181Cys chr17: 7578389G>A	rs587782596	1
	exon8: c.802_803del: p.Asn268GlnfsTer3		1
	exon6: c.573del: p.Gln192SerfsTer55		1
	exon4:c.150_151insT: p.Glu51Ter		1
	exon5: c.488A>G: p.Tyr163Cys		1
	exon7: c.730G>A: p.Gly244Ser		1
	c.673-10_675del chr17: 7577606_7577618del		1
	exon5: c.541C>T: p.Arg181Cys		1
	c.637C>T: p.(Arg213*) chr177:7578212G>A	rs397516436	1
	c.797G>T p.Gly266Val chr17: 7577141C>A	rs193920774	1
	exon4: c.192del: p.Arg65fs		1
BRCA1	exon10: c.1285delA: p.Ile429Ter		1
	exon23: c.5497G>A p.Val1833Met		1
	exon6: c.427del: p.Glu143LysfsTer20		1
	exon13: c.4467delA: p.Glu1490AsnfsTer15		1
	exon2: c.75_76ins: p.Ile26SerfsTer16)		1
	exon4: c.181T>G: p.Cys61Gly	rs28897672	1
	c.5293 G>T p.Glu1765*		1
	c.5213_5278-2753delinsT exon20		1
	c.5266dupC p.Gln1756Profs*74		1
	c.2923C>T p.Gln975*		1
	exon 10 c.3679C>T p.Gln1227Ter		1
RB1	exon20: c.2077 G>T: p.Glu693Ter		1
NF1	c.6084+2T>G chr17: 29663493T>G		1
BRCA2	c.3554_3563del p.Thr1185Ilefs*9		1
AKT1	exon4: c.235C>A p.Gln79Lys		1
RAD50	exon3: c.326_329del: p.Thr109AsnfsTer20		1
PIK3CA	exon10: c.1633 G>A: p.Glu545Lys		1

The number of cases that each genetic polymorphism was identified is marked as frequency.

Variants of unknown significance (VUS) somatic mutations are presented in **Table 2**. The most common genes with VUS somatic mutations in HGSC were ROS1 [26%; 13/50], RAD50 [6%; 3/50], BRCA2 [6%; 3/50], NOTCH1 [6%; 3/50], TP53 [6%; 3/50], AR [6%; 3/50]. Other genes with VUS mutations identified include CCNE1, MEN1, TP53, PTEN, ATM, FGFR3, MSH2, MYC, IDH1, KMT2C, RAD51, CHEK2, JAK2, KDR, PALB2, BCL2, CHECK1, MSH6, CTNNB1, PDGFRA STK11, BRCA1.

**Table 2:** Somatic mutations of uncertain significance (VUS) identified in patients with HGSC.

Gene mutation	Genetic polymorphism	NCBI Genomes Browser	Frequency
ROS1	exon1: c.49C>G: p.Leu17Val		1
	exon43: c.6797C>T p.Thr2266Met		1
	exon5: c.433A>C: p.Thr145Pro		5
	exon6: c.500G>A: p.Arg167Gln		4
	exon12: c.1519A>G: p.Asn507Asp		1
	exon8: c.799A>G: p.Asn267Asp		1
	exon12: c.1538 A>T: p.Asp513Val		1
	exon41: c.4484T>G p.Tyr2162Asp		1
CCNE1	c.779A>T: p.Asn260Ile chr19: 30312976A>T	rs61750863	1
MEN1	c.541G>T: p.Ala181Ser chr11: 64575491C>A	rs376872829	1
	c.563C>T: p.Pro188Leu chr6: 64575454G>A	rs199706698	1
TP53	exon7: c.721del: p.Ser241fs		1
	exon5: c.552_553del: p.Asp184fs		1
	exon7: c.685_690del p.Cys229_Thr230del		1
PTEN	exon5: c.365T>G: p.Ile122Ser		1
ATM	exon12: p.Leu612Pro c.1835T>C		11
FGFR3	exon5: c.560C>A: p.Ser187Tyr		1
MSH2	exon11: c.1681G>A: p.Glu561Lys		1
	c.1045C>G p.Pro349Alachr2: 47643537C>G	rs267607939	1
NOTCH1	c.4103G>A: p.Arg1368His chr9: 139400245C>T (hg19)	rs779086531	1
	exon21: c.3395G>A: p.Arg1132His		1
	exon14: c.2353G>A: p.Gly785Ser		1
MYC	c.737C>T: p.Pro246Leu chr8: 128751200C>T	rs545330879	1
IDH1	c.388A>G: p.Ile130Val chr6: 209113119T>C	rs762479277	1
KMT2C	c.943G>T: p.Gly315Cys chr7: 151970859 C>A	rs149992209	1
	exon56: c.14358 T>G: p.Tyr4786Ter		1
AR	c.158G>A: p.Ser53Asn chrX: 66765146G>A		1
	c.1208 C>T: p.Ala403Val chrX: 66766196 C>T	rs772490323	1
	exon1: c.1174 C>T: p.Pro392Ser		1
RAD51	c.707G>A: p.Arg236Gln chr15: 41021762 G>A		1
	c.197C>T p.Thr66Met		1
RAD50	c. 980 G>A p.R327H chr5: 131923710 G>A	rs28903091	1
	c.130A>T: p.Thr44Ser chr5:131894976A>T	rs377388354	1
	c.1636-3T>G chr5:131927566T>G		1
CHEK2	c.320-5T>A chr22: 29121360 A>T	rs121908700	1
BRCA2	exon11: c.4412_4414del: p.Arg1471del		1
	exon11: c.3985A>G: p.Arg1329Gly		2
JAK2	exon3: c.143G>A: p.Gly48Glu		1
KDR	exon14: c.2012G>A: p.Gly671Glu		1
PALB2	exon13: c.3428T>A: p.Leu1143His		1
BCL2	exon2: c.119_120del: p.Pro40ArgfsTer112		1
CHECK1	exon10: c.1040C>A p.Pro347His		1
MSH6	exon5: c.3256C>A: p.Pro1086Thr		1
BRCA1	exon10: c.3367G>T: p.Asp1123Tyr		1
MYC1	exon2: c.77A>G: p.Asn26Ser		1
CTNNB1	exon12: c.1907C>T: p.Ala636Val		1
FANCL	c.203G>c p.Arg68Pro		1
PDGFRA	exon22: c.3082G>T p.Val1028Phe		1
STK11	c.911G>A p.Arg304Glnchr19:1221996G>A hg	rs376280361	1

Germline NGS analysis was performed in 30 patients with HGSC (**Supplemental Table 2**). All germline mutations are presented in **Table 3**. The most frequent mutated genes identified were BRCA1 [8/30; 27%] and BRCA2 [4/30; 13%] as expected. Other pathogenic germline mutations were identified in APC (c.3920T>A) and MUTYH (c.452A>G) genes. Of note, the most common VUS mutations were identified in HRR-related genes, including ATM (c.7816A>G), BRIP (c.2327 C>A), CHEK2 (c.320-5T>A).

**Table 3:** Germline mutations in patients with HGSC.

Gene	Genetic polymorphism	NCBI Genomes Browser	Clinical Significance	Frequency
BRCA2	c.1405_1406del p.Asp469* chr 13.32907016_32907017del	rs397507586	Pathogenic	1
	c.2339C>G p.Ser780* chr13: 32910831 C>G	rs587781471	Pathogenic	1
	c.3554_3563del p.Thr1185Ilefs*9 chr13: 32912046_32912055del	rs397507675	Pathogenic	1
	c.2808_2811del: p.Ala938Profs*21 chr13: 32911300_32911303del	rs80359351	Pathogenic	1
	c.7355 A>T p.Asn2452Ile chr13: 32929345 A>T		VUS	1
	c.8117A>G p.Asn2706Ser chr13: 32937456 A>G	rs80359055	VUS	1
BRCA1	c.5497G>A p.Val1833Met chr17:41197790 C>T	rs80357268	Pathogenic	2
	exon6: c.427del: p.Glu143LysfsTer20		Pathogenic	1
	exon20: c.5194-452_5277+3638del NM_007294.3		Pathogenic	1
	c.3679C>T p.Gln1227Ter		Pathogenic	1
	c.3481_3491del: p.Glu1161Phefs exon11		Pathogenic	1
	c.3157dup p.Glu1053Glyfs*7 chr17: 41244391 dup	rs397509042	Pathogenic	1
	p.Cys61Gly c.181 T>G		Pathogenic	1
	c.3541G>A p.Val1181Ile	rs56336919	VUS	1
	c.457A>G p.Ser153Gly		VUS	1
	c.536A>G p.Tyr179Cys		VUS	1
	c.1456T>C p.Phe486Leu		VUS	1
	c.1648A>C p.Asn550His		VUS	1
	c.3367G>T p.Asp1123Tyr		VUS	1
APC	c.3920T>A p.Ile1307Lys chr5:112175211 T>A	rs1801155	Pathogenic	1
MUTYH	c.452A>G p.Tyr151Cys		Pathogenic	1
MLH1	c.1460G>A (p.Arg487Gln) chr3: 37070325G>A	rs587778917	VUS	1
MSH2	c.1043A>G p.Gln348Arg chr2: 47643535 A>G	rs773177076	VUS	1
	c.1321 A>T p.Thr441Ser chr2: 47672731 A>T		VUS	1
	c.439 G>A p.Val147Ile chr2: 47637305 G>A	rs773125415	VUS	1
ATM	c.7816A>G p.Ile2606Val chr11: 108203516 A>G	rs376824528	VUS	1
BRIP1	c.2327 C>A p.Ala776Asp chr17: 59820426 G>T	rs1555590421	VUS	1
CHEK2	c.320-5T>A chr22: 29121360 A>T	rs121908700	VUS	1

### Other histologies

All variants identified in patients with LGSC are summarized in **Table 4**. Somatic mutations in BRCA1(c.5497G>A) and NRAS (c.182A>G) genes were detected. Although very uncommon, one patient with LGSC harbored a TP53 gene mutation (c.818G>A). In endometrioid carcinoma [EC], PTEN [3/7; 43%] was the most common gene with pathogenic mutations (**Table 5**). Pathogenic mutations in other genes include ATM (c.3539_3540del), PIK3CA (c.1633G>A), MSH6 (c.2342del), KMT2C (c.5053G>T) and KRAS (c.37G>T). In OCCC, PIK3CA (c.1637A>G) and KRAS (c.35G>T) (**Table 6**) were the two genes with pathogenic mutations. Two patients with mucinous carcinomas were analyzed. TP53 (c.818G>A) pathogenic variant and somatic VUS in IDH1 (c.548A>G), STK11 (c.901C>T) and ROS1 (c.6344A>G, c.6098A>C, c.1094G>C) genes were identified in these patients.

**Table 4:** Somatic mutations in patients with LGSC.

Subject	Pathogenic somatic mutations	Somatic VUS
1	**BRCA1** (exon23: c.5497G>A p.Val1833Met) **NRAS** (exon3: c.182A>G p.Gln61Arg)	–
2	–	–
3	–	**ROS1** (c.1816_1824del: p.Gln606_Trp608del chr6: 117709132_117709141delinsT (hg19) rs748934319) **KDR** (c.475G>A: p.Val159Met chr4: 55981462C>T (hg19) rs545112274)
4	**TP53** (exon8: c.818G>A: p.Arg273His)	**ROS1** (exon12: c.1611A>G p.Ile537Met)
5	**-**	**-**
6	**-**	**-**
7	**-**	**-**

**Table 5:** Somatic mutations in patients with endometrioid (EC) ovarian carcinoma.

Subject	Pathogenic somatic mutations	Somatic VUS
1	–	**ATM** (c.8428A>C p.Lys2810Gln) chr11: 108216479 A>C (hg19) rs730881325)
2	–	–
3	**ATM** (c.3539_3540del: p.Val1180Glufs*19 chr11: 108151858_108151859del (hg19)) **PTEN** (c.202T>C: p.Tyr68His chr10: 89685307 T>C rs398123317)	**ATM** (c.8672 G>A p.Gly2891Asp chr11: 108224493 G>A (hg19) rs748192003) **FGFR2** (c.1840A>G: p.Met614Val chr10: 123256069 T>C (hg19) rs751047267) **PTEN** (c.141G>T: p.Arg47Ser chr10: 89653843 G>T) **MAP2K1** (c.106_108del: p.Lys36del chr15: 66727390_66727392del (hg19))
4	**MSH6** (exon4: c.2342del: p.Pro781HisfsTer12) **MYC** (exon2: c.176C>T: p.Ala59Val) **KMT2C** (exon34: c.5053G>T: p.Ala1685Ser) **CCND1** (exon5: c.859C>T p.Pro287Ser) **PTEN** (exon2: c.121del: p.Arg41AspfsTer13) **PTEN** (exon7: c.752G>T: p.Gly251Val) **KRAS** (exon2: c.37G>T: p.Gly13Cys)	**KDR** (exon10: c.1325 C>T: p.Thr442Met)
5	**PIK3CA** (exon10: c.1633G>A p.Glu545Lys) **PTEN** (exon3: c.202T>C p.Tyr68His)	–
6	**TP53** (exon5: c.501delG p.Gln167HisfsTer3)	–
7	**-**	**-**

**Table 6:** Somatic mutations in patients with OCCC.

Subject	Pathogenic somatic mutations	Somatic VUS
1	**PIK3CA** (exon10: c.1637A>G: p.Gln546Arg) **KRAS** (exon2: c.35G>T: p.Gly12Val)	–
2	–	**KMT2C** (exon38: c.9245C>T: p.Pro3082Leu)
3	–	–
4	–	–

## Discussion

We here report the mutational landscape of ovarian cancer patients treated in the Oncology Department of Alexandra University Hospital as defined that underwent powerful NGS analysis. In the era of precision medicine, the identification of druggable mutations could help to optimize treatment especially after the introduction of PARP inhibitors in clinical practice. Mutations in HRD genes other than BRCA1/2 that are necessary for successful HR repair (RAD50, RAD51, ATM, PALB2, RAD52, ATM, CHEK2) can lead to genomic instability and HR deficiency. Of note, one in four women with ovarian cancer will have a germline HR deficiency and an additional 5–7% will have a somatic HR deficiency (9).

Patients with HGSC comprise the main population in our analysis. TP53 [68%; 34/50] and BRCA1 [22%; 11/50] ARE the genes with the highest number of pathogenic somatic mutations, as described in previous literature. However, many other genes with pathogenic somatic mutations were also reported (RB1, NF1, BRCA2, AKT1, RAD50, PIK3CA). It has been described that recurrent mutations in NF1 (4-6%), RB1 (2-6%) and PTEN (<1%) are observed in HGSC and when including larger deletions these alterations become even more frequent (NF1: 20%; RB1: 17%; PTEN: 7%) (10). In accordance with these data, pathogenic mutations in RB1 [2%; 1/50] and NF1 [2%; 1/50] were also identified in our study. In addition, pathogenic and VUS mutations involve genes of the homologous recombination (HR) pathway. These genes include ATM, RAD51, CHEK2, PALB2, CHECK1, CTNNB1, BRCA1. As it is known, alterations in HRR pathway genes harbor the 50% of HGSC tumors. HRR alterations involve Fanconi anemia genes (PALB2, FANCA, FANCL, FANCI, FANCC), core RAD genes (RAD50, RAD51, RAD51B, RAD51C) as well as DNA damage response genes (ATM, ATR, CHEK1, CHEK2) (10). The Cancer Genome Atlas (TCGA) analysis reported that HGSC tumors are characterized by mutations in PTEN (7%), RAD51C (3%), ATM/ATR (2%) and Fanconi anemia genes (5%). Of note, Pennington et al. associated the presence of somatic or germline HR mutations with platinum sensitivity and favorable prognosis (P = 0.0006) (11). Overall survival was 59 months for patients with somatic HR mutations in contrast to 41 months of non-carriers (11).

The majority of patients were characterized by BRCA1 [8/30; 27%] and BRCA2 [4/30; 13%] germline mutations, while VUS germline mutations were identified in HRR-related genes, including ATM (c.7816A>G), BRIP (c.2327 C>A), CHEK2 (c.320-5T>A). Another study also reported similar findings (11). Apart from BRCA1/2 germline mutations that predominate in HGSC, other germline mutations were reported in HR genes: (2%) in *BARD1*, 4 (4.5%) in *BRIP1*, 1 (1%) in *CHEK1*, 3 (3%) in *CHEK2*, 2 (2%) in *FAM175A*, 1 (1%) in *NBN*, 2 (2%) in *PALB2*, 3 (3%) in *RAD51C* and 4 (4.5%) in *RAD51D.* Recent literature supports that up to 24% of ovarian cancers are associated with germline mutations, and of these, 29% harbor mutations in genes other than BRCA1/2 (9). Germline mutations in other HRR genes including BRIP1, ATM RAD51C, RAD51D contribute cumulatively to about 2% of HGSC (10). These genetic alterations are both prognostic and predictive biomarkers: HRD tumors are associated with a prolonged survival, sensitivity to platinum chemotherapy and PARP inhibition (10, 11). We also report one case of germline pathogenic APC (c.3920T>A) mutation and another of germline pathogenic MUTYH (c.452A>G) mutation. Heterozygous APC germline mutations predispose to familial adenomatous polyposis (FAP) syndrome and a lifetime risk for colorectal cancer (12). APC germline mutation will lead to the development of more than 100 adenomas at adolescence that will eventually lead to CRC by the age of 40 if left untreated (12). Similarly, MUTYH germline mutations cause an autosomal recessive form of familial adenomatous polyposis known as MYH-associated polyposis (MAP) (13). People with MAP tend to develop multiple adenomatous colon polyps although sometimes at a lesser extent than those with FAP (10-100 adenomas) (13). Approximately 1 to 2 percent of the general population carry a single (monoallelic) germline *MUTYH* pathogenic variant, whereas biallelic *MUTYH* pathogenic variants are found in 7 to 13 percent of patients with >100 adenomas and in 14 to 40 percent of patients with 10 to 99 adenomas. Interestingly, MAP is associated with the significant increase in the incidence of ovarian, bladder, and skin cancers and a trend of increased risk of breast cancer (14). There are also reports of APC carriers developing ovarian or endometrial tumors although rarely (15). Our patient had a synchronous BRCA1 germline mutation which explains the development of HGSC ovarian cancer.

Focusing on non-high grade histologies, BRCA1(c.5497G>A) and NRAS (c.182A>G pathogenic somatic mutations were detected in LGSC patients. According to literature, LGSC commonly involves mutations in MAPK pathway genes (KRAS [25%], BRAF [8%], NRAS [8%]), whereas BRCA1/2 are rarely detected (10). In EC patients, PTEN [3/7; 43%] was the most common pathogenic mutations identified, along with ATM, PIK3CA, MSH6, KMT2C and KRAS mutations. Similarly, PIK3CA and KRAS were the main pathogenic mutations identified in OCCC. Based on a dualistic model of carcinogenesis, EOC could be divided into two categories: type I tumors that comprise of LGSC, low-grade EC, OCCC and mucinous carcinomas and type II tumors that include HGSC, high-grade EC and undifferentiated carcinomas. Type I tumors are relatively genetically stable and are characterized by specific mutations, including KRAS, BRAF, ERBB2, CTNNB1, PTEN PIK3CA, ARID1A and PPPR1A, but rarely harbor TP53 mutations (16). Indeed, the most common mutations observed in EC are CTNNB1 [30-50%], PIK3CA [15-40%], ARID1A [30-35%], PTEN [20-30%] and KRAS [10-30%] (10). OCCC significantly overlaps with EC in terms of mutational profile harboring ARID1A [50-75%], PIK3CA [40-50%] and KRAS [15%] in the vast majority of cases (10). Both histological subtypes typically arise from endometriosis and are the predominant subtypes observed in patients with endometriosis (60% for EC and 15% for OCCC). These data come in agreement with the mutations identified in our study, although the number of cases is relatively limited.

Concerning the limitations of our study that deserve mention, it is important to advert that we analyzed a small number of tumor samples and blood samples due to the increased cost of sequencing. Despite the small number of samples, NGS is highly accurate and rapidly analyzes the targeted regions of the genome enabling the characterization of both pathogenic and VUS mutations. Given the fact that most ovarian cancer patients undergo either a more targeted genetic testing e.g. BRCA1/2 gene test or an only somatic mutation test, this genome-wide analysis is of high value.

We here report the mutational profile of ovarian cancer patients treated in a single institution. Germline and somatic tumor genetic testing should be offered in most patients with ovarian carcinomas as a prognostic and predictive biomarker. Since the approval of novel therapies including PARP inhibitors in clinical practice, we should identify the population that could be of the utmost benefit from this treatment.

## Data availability statement

The original contributions presented in the study are included in the article/**Supplementary Material**. Further inquiries can be directed to the corresponding author.

## Ethics statement

The studies involving human participants were reviewed and approved by Institutional Review Board of Alexandra University Hospital (Ethics code: 513/15-07-2020). The patients/participants provided their written informed consent to participate in this study.

## Author contributions

FZ, KK, and M-AD: Conceptualization, KA: validation, KA: formal analysis, AA, A-MP, AK. MK. and EZ; investigation, AA, A-MP, AK, EZ, and MK: data curation, AA; writing—original draft preparation, AA, AK, ML, FZ, and EZ: writing—review and editing, A-MP, FZ, KK, and M-AD: supervision, FZ, M-AD, KK, and ML: project administration. All authors have read and agreed to the published version of the manuscript.

## Conflict of interest

KK has received honoraria by Roche, BMS, MSD and IPSEN. M-AD has received honoraria from participation in advisory boards from Amgen, Bristol-Myers-Squibb, Celgene, Janssen, Takeda. FZ has received honoraria for lectures and has served in an advisory role for Astra-Zeneca, Daiichi, Eli-Lilly, Merck, Novartis, Pfizer, and Roche. ML has received honoraria by Roche, Astra Zeneca, Astellas, MSD, Janssen, BMS and IPSEN.

The remaining authors declare that the research was conducted in the absence of any commercial or financial relationships that could be construed as a potential conflict of interest.

## Publisher’s note

All claims expressed in this article are solely those of the authors and do not necessarily represent those of their affiliated organizations, or those of the publisher, the editors and the reviewers. Any product that may be evaluated in this article, or claim that may be made by its manufacturer, is not guaranteed or endorsed by the publisher.
