# Supplementary material for: Germline and somatic variants in ovarian carcinoma: A next-generation sequencing (NGS) analysis
Source: Front Oncol. 2022 Dec 1;12:1030786. doi: 10.3389/fonc.2022.1030786 (PMC9754718; doi:10.3389/fonc.2022.1030786)
Supplement: Supplementary file 1 [file DataSheet_1.docx]

**SUPPLEMENTARY MATERIAL**

| AKT1 | BRCA1 | CDKN2A | ERBB4 | IDH2 | KRAS | MLH1 | NOTCH1 | PTEN | SMAD4 |
| --- | --- | --- | --- | --- | --- | --- | --- | --- | --- |
| ALK | BRCA2 | CHEK1 | ESR1 | JAK2 | MAP2K1 | MSH2 | NRAS | RAD50 | STAT3 |
| AR | CCND1 | CHEK2 | FGFR1 | JUN | MDM2 | MSH6 | PALB2 | RAD51 | STK11 |
| ATM | CCNE1 | CTNNB1 | FGFR2 | KDR | MDM4 | mTOR | PDGFRA | RB1 | TP53 |
| BCL2 | CDK4 | EGFR | FGFR3 | KIT | MEN1 | MYC | PIK3CA | RET |  |
| BRAF | CDK6 | ERBB2 | IDH1 | KMT2C | MET | NF1 | PIK3CB | ROS1 |  |

**Supplementary Table 1**. Gene panel tested in FFPE tissues

| APC | ATM | BAP1 | BLM | BMPR1A | BRCA1 | BRCA2 | BRIP1 | CDH1 | CDKN2A | |
| --- | --- | --- | --- | --- | --- | --- | --- | --- | --- | --- |
| CHEK2 | DICER1 | FANCM | FH | FLCN | MEN1 | MLH1 | MSH2 | MSH6 | MUTYH | |
| NBN | NF1 | NF2 | PALB2 | PMS1 | PMS2 | PTEN | RAD51C | RAD51D | RB1 | |
| RECQL4 | RET | SDHB | SDHC | SDHD | SLX4 | SMAD4 | STK11 | TP53 | TSC1 | |
| TSC2 | VHL |  |  |  |  |  |  |  |  |  |

**Supplementary Table 2**. Gene panel analyzed in the germline NGS study

| Subject | Pathogenic somatic mutations | Variants of unknown significance (VUS) |
| --- | --- | --- |
| 1 | **TP53** (exon8: c.818G>A p.Arg273His) | - |
| 2 | **BRCA1** (exon10: c.1285delA: p.Ile429Ter)  **TP53** (exon7: c.764_766del: p.Ile255del) | - |
| 3 | **TP53** (exon5: c.517G>A: p.Val173Met) | **ROS1** (exon1: c.49C>G: p.Leu17Val) |
| 4 | **TP53** (c.586C>T: p.Arg196*) chr17: 7578263G>A (hg19) rs397516435 | **CCNE1** (c.779A>T: p.Asn260Ile chr19: 30312976A>T rs61750863)  **MEN1** (c.541G>T: p.Ala181Ser chr11: 64575491C>A rs376872829) |
| 5 | **TP53** (c.614A>G p.Tyr205Cys chr17: 7578235 T>C rs1057520007) | - |
| 6 | **BRCA1** (exon23: c.5497G>A p.Val1833Met) | **TP53** (exon7: c.721del: p.Ser241fs)  **PTEN** (exon5: c.365T>G: p.Ile122Ser) |
| 7 | **TP53** (exon7: c.734G>T: p.Gly245Val) | **ROS1** (exon43: c.6797C>T p.Thr2266Met) |
| 8 | - | **ATM** (exon12: p.Leu612Pro c.1835T>C)  **ROS1** (exon5: c.433A>C: p.Thr145Pro) |
| 9 | **BRCA1** (exon6: c.427del: p.Glu143LysfsTer20)  **TP53** (exon5: c.524G>A p.Arg175His) | - |
| 10 | **TP53** (exon8: c.840A>C: p.Arg280Ser) | **ROS1** (exon5: c.433A>C: p.Thr145Pro)  **FGFR3** (exon5: c.560C>A: p.Ser187Tyr) |
| 11 | **BRCA1** (exon13: c.4467delA: p.Glu1490AsnfsTer15) | **ROS1** (exon6: c.500G>A: p.Arg167Gln) |
| 12 | - | **MSH2** (exon11: c.1681G>A: p.Glu561Lys)  **ROS1** (exon12: c.1519A>G: p.Asn507Asp)  **ROS1**(exon8: c.799A>G: p.Asn267Asp) |
| 13 | - | - |
| 14 | - | - |
| 15 | - | **NOTCH1** (c.4103G>A: p.Arg1368His chr9: 139400245C>T (hg19) rs779086531)  **MYC** (c.737C>T: p.Pro246Leu chr8: 128751200C>T (hg19) rs545330879) |
| 16 | **TP53** (c.796G>C: p.Gly266Arg chr17: 7577142C>G (hg19) rs1057519990) | **IDH1** (c.388A>G: p.Ile130Val chr6: 209113119T>C (hg19) rs762479277)  **KMT2C** (c.943G>T: p.Gly315Cys chr7: 151970859 C>A (hg19) rs149992209)  **AR** (c.158G>A: p.Ser53Asn chrX: 66765146G>A (hg19)) |
| 17 | **TP53** (c.1024del: p.Arg342Glufs*3 chr17: 7574003del (hg19)) | **RAD51** (c.707G>A: p.Arg236Gln chr15: 41021762 G>A (hg19)) |
| 18 | **TP53** (exon6: c.659 A>G: p.Tyr220Cys) | - |
| 19 | **TP53** (c.673-1G>A chr17:7577609 C>T (hg19) rs878854073) | **RAD50** (c. 980 G>A p.R327H chr5: 131923710 G>A (hg19) rs28903091) |
| 20 | **BRCA1** (exon2: c.75_76ins: p.Ile26SerfsTer16)  **TP53** (exon4: c.192del: p.Arg65fs) | **ROS1** (exon12: c.1538 A>T: p.Asp513Val)  **KMT2C** (exon56: c.14358 T>G: p.Tyr4786Ter) |
| 21 | **BRCA1** (exon4: c.181T>G: p.Cys61Gly rs28897672)  **RB1** (exon20: c.2077 G>T: p.Glu693Ter) | **TP53** (exon5: c.552_553del: p.Asp184fs)  **BRCA2** (exon11: c.4412_4414del: p.Arg1471del) |
| 22 | **TP53** (exon7: c.742 C>T: p.Arg248Trp) | - |
| 23 | **TP53** (exon8: c.833 C>T: p.Pro278Leu)  **PIK3CA** (exon10: c.1633 G>A: p.Glu545Lys) | **ROS1** (exon6: c.500G>A: p.Arg167Gln) |
| 24 | **BRCA1** c.5293 G>T p.Glu1765*)  **BRCA2** (c.3554_3563del p.Thr1185Ilefs*9) | **RAD51** (c.197C>T p.Thr66Met) |
| 25 | **TP53** (c.637 C>T: p.Arg213* chr17: 7578212 G>A (hg19) rs397516436)  **NF1** (c.6084+2T>G chr17: 29663493T>G (hg19)) | **CHEK2** (c.320-5T>A chr22: 29121360 A>T (hg19) rs121908700)  **AR** (c.1208 C>T: p.Ala403Val chrX: 66766196 C>T (hg19) rs772490323) |
| 26 | **TP53** (exon4: c.245del: p.Pro82ArgfsTer41) | **AR** (exon1: c.1174 C>T: p.Pro392Ser) |
| 27 | **BRCA1** (c.5213_5278-2753delinsT exon20) | - |
| 28 | **TP53** (exon6: c.658T>A: p.Tyr220Asn) | - |
| 29 | **TP53** (exon5: c.438G>A: p.Trp146Ter) | **JAK2** (exon3: c.143G>A: p.Gly48Glu)  **BRCA2** (exon11: c.3985A>G: p.Arg1329Gly) |
| 30 | **BRCA1** (c.5266dupC p.Gln1756Profs*74) | - |
| 31 | **TP53** (exon5: c.524G>A: p.Arg175His) | **ROS1** (exon5: c.433 A>C: p.Thr145Pro) |
| 32 | **TP53** (exon7: c.742del: p.Arg248GlyfsTer97) | **ROS1** (exon6: c.500G>A: p.Arg167Gln)  **ROS1** (exon5: c.433A>C: p.Thr145Pro)  **KDR** (exon14: c.2012G>A: p.Gly671Glu) |
| 33 | **TP53** (exon8: c.818G>A p.Arg273His) | **PALB2** (exon13: c.3428T>A: p.Leu1143His)  **BCL2** (exon2: c.119_120del: p.Pro40ArgfsTer112)  **NOTCH1** (exon21: c.3395G>A: p.Arg1132His) |
| 34 | **TP53** (exon10: c.997delC p.Arg333ValfsTer12) | - |
| 35 | **TP53** (c.541C>T p.Arg181Cys chr17: 7578389G>A (hg19) rs587782596) | **RAD50** (c.130A>T: p.Thr44Ser chr5:131894976A>T (hg19) rs377388354) |
| 36 | **TP53** (exon8: c.802_803del: p.Asn268GlnfsTer3) | **CHECK1** (exon10: c.1040C>A p.Pro347His)  **MSH6** (exon5: c.3256C>A: p.Pro1086Thr) |
| 37 | **BRCA1** (c.2923C>T p.Gln975*) | - |
| 38 | **TP53** (exon6: c.573del: p.Gln192SerfsTer55) | **NOTCH1** (exon14: c.2353G>A: p.Gly785Ser)  **BRCA1** (exon10: c.3367G>T: p.Asp1123Tyr) |
| 39 | **BRCA1** (exon 10 c.3679C>T p.Gln1227Ter)  **TP53** (exon4: c.150_151insT: p.Glu51Ter) | **ROS1** (exon6: c.500G>A p.Arg167Gln) |
| 40 | **-** | - |
| 41 | **TP53** (c.524G>A: p.Arg175His chr17: 7578406C>T (hg19) rs28934578) | - |
| 42 | **TP53** (exon5: c.488A>G: p.Tyr163Cys) | **MYC1** (exon2: c.77A>G: p.Asn26Ser)  **CTNNB1** (exon12: c.1907C>T: p.Ala636Val) |
| 43 |  | **FANCL** (c.203G>c p.Arg68Pro) |
| 44 | **TP53** (exon7: c.730G>A: p.Gly244Ser) | **PDGFRA** (exon22: c.3082G>T p.Val1028Phe) |
| 45 | **TP53** (c.673-10_675del chr17: 7577606_7577618del (hg19) | - |
| 46 | **-** | **RAD50** (c.1636-3T>G  chr5:131927566T>G hg(19))  **STK11** (c.911G>A p.Arg304Gln  chr19:1221996G>A hg(19)  rs376280361)  **MSH2** (c.1045C>G p.Pro349Ala  chr2: 47643537C>G hg(19)  rs267607939) |
| 47 | **TP53** (c.797G>T p.Gly266Val chr17: 7577141C>A (hg19) rs193920774) | **-** |
| 48 | **TP53** (c.637C>T: p.(Arg213*) chr177:7578212G>A (hg19) rs397516436) | **MEN1** (c.563C>T: p.Pro188Leu chr6: 64575454G>A (hg19) rs199706698) |
| 49 | **TP53** (exon5: c.541C>T: p.Arg181Cys)  **RAD50** (exon3: c.326_329del: p.Thr109AsnfsTer20) | **BRCA2** (exon11: c.3985A>G p.Arg1329Gly)  **ROS1** (exon5: c.433A>C p.Thr145Pro) |
| 50 | **AKT1** (exon4: c.235C>A p.Gln79Lys) | **TP53** (exon7: c.685_690del p.Cys229_Thr230del)  **ROS1** (exon41: c.4484T>G p.Tyr2162Asp) |

***Supplemental Table 3.*** Pathogenic and VUS somatic mutations identified in 50 patients with HGSC

| Subject | Pathogenic germline mutations | Variants of unknown significance (VUS) germline |
| --- | --- | --- |
| 1 | **BRCA2** (c.1405_1406del p.Asp469* chr 13.32907016_32907017del (hg19) rs397507586 | **MLH1** (c.1460G>A (p.Arg487Gln) chr3: 37070325G>A (hg19) rs587778917) |
| 2 | - | - |
| 3 | **MUTYH** (c.452A>G p.Tyr151Cys) | - |
| 4 | - | **MSH2** (c.1043A>G p.Gln348Arg chr2: 47643535 A>G rs773177076) |
| 5 | **BRCA1** (c.5497G>A p.Val1833Met chr17:41197790 C>T rs80357268)  **APC** (c.3920T>A p.Ile1307Lys chr5:112175211 T>A rs1801155) | - |
| 6 | **BRCA1** (exon6: c.427del: p.Glu143LysfsTer20) | - |
| 7 | - | - |
| 8 | - | **BRCA2** (c.8117A>G p.Asn2706Ser chr13: 32937456 A>G (hg19) rs80359055) |
| 9 | - | - |
| 10 | **BRCA2** (c.2339C>G p.Ser780* chr13: 32910831 C>G (hg19) rs587781471) | - |
| 11 | - | **ATM** (c.7816A>G p.Ile2606Val chr11: 108203516 A>G (hg19) rs376824528) |
| 12 | - | - |
| 13 | - | **MSH2** (c.1321 A>T p.Thr441Ser chr2: 47672731 A>T (hg19) |
| 14 | **BRCA1** (p.Cys61Gly c.181 T>G) |  |
| 15 | - | **MSH2** (c.439 G>A p.Val147Ile chr2: 47637305 G>A (hg19) rs773125415) |
| 16 | **BRCA2** (c.3554_3563del p.Thr1185Ilefs*9 chr13: 32912046_32912055del (hg19) rs397507675) | - |
| 17 | - | **BRCA2** (c.7355 A>T p.Asn2452Ile chr13: 32929345 A>T (hg19))  **BRIP1** (c.2327 C>A p.Ala776Asp chr17: 59820426 G>T (hg19) rs1555590421) |
| 18 | - | **CHEK2** (c.320-5T>A chr22: 29121360 A>T (hg19) rs121908700) |
| 19 | - | - |
| 20 | **BRCA1** (c.5497G>A p.Val1833Met rs80357268) | - |
| 21 | **BRCA1** (exon20: c.5194-452_5277+3638del NM_007294.3) | - |
| 22 | **BRCA1** (c.3481_3491del: p.Glu1161Phefs exon11) |  |
| 23 | **BRCA2** (c.2808_2811del: p.Ala938Profs*21 chr13: 32911300_32911303del (hg19) rs80359351) | - |
| 24 | **BRCA1** (c.3157dup p.Glu1053Glyfs*7 chr17: 41244391 dup (hg19) rs397509042) | - |
| 25 | - | - |
| 26 | - | **BRCA1** (c.457A>G p.Ser153Gly) |
| 27 | **BRCA1** (c.3679C>T p.Gln1227Ter) | - |
| 28 | - | **BRCA1** (c.3541G>A p.Val1181Ile rs56336919) |
| 29 | - | **BRCA1** (c.536A>G p.Tyr179Cys)  **BRCA1** (c.1456T>C p.Phe486Leu)  **BRCA1** (c.1648A>C p.Asn550His)  **BRCA1** (c.3367G>T p.Asp1123Tyr) |
| 30 | - | - |

***Supplemental Table 4.*** Pathogenic and VUS germline mutations identified in 30 patients with HGSC
